# Evaluation of Approaches to Monitor *Staphylococcus aureus* Virulence Factor Expression during Human Disease

**DOI:** 10.1371/journal.pone.0116945

**Published:** 2015-02-26

**Authors:** Wouter Rozemeijer, Pamela Fink, Eduardo Rojas, C. Hal Jones, Danka Pavliakova, Peter Giardina, Ellen Murphy, Paul Liberator, Qin Jiang, Douglas Girgenti, Remco P. H. Peters, Paul H. M. Savelkoul, Kathrin U. Jansen, Annaliesa S. Anderson, Jan Kluytmans

**Affiliations:** 1 VU University Medical Center, Amsterdam, The Netherlands; 2 Pfizer Vaccine Research and Development, Pearl River, New York, United States of America; Universitätsklinikum Hamburg-Eppendorf, GERMANY

## Abstract

*Staphylococcus aureus* is a versatile pathogen of medical significance, using multiple virulence factors to cause disease. A prophylactic *S. aureus* 4-antigen (SA4Ag) vaccine comprising capsular polysaccharide (types 5 and 8) conjugates, clumping factor A (ClfA) and manganese transporter C (MntC) is under development. This study was designed to characterize *S. aureus* isolates recovered from infected patients and also to investigate approaches for examining expression of *S. aureus* vaccine candidates and the host response during human infection. Confirmation of antigen expression in different disease states is important to support the inclusion of these antigens in a prophylactic vaccine. Hospitalized patients with diagnosed *S. aureus* wound (27) or bloodstream (24) infections were enrolled. Invasive and nasal carriage *S. aureus* isolates were recovered and characterized for genotypic diversity. *S. aureus* antigen expression was evaluated directly by real-time, quantitative, reverse-transcriptase PCR (qRT-PCR) analysis and indirectly by serology using a competitive Luminex immunoassay. Study isolates were genotypically diverse and all had the genes encoding the antigens present in the SA4Ag vaccine. *S. aureus* nasal carriage was detected in 55% of patients, and in those subjects 64% of the carriage isolates matched the invasive strain. In swab samples with detectable *S. aureus* triosephosphate isomerase housekeeping gene expression, RNA transcripts encoding the *S. aureus* virulence factors ClfA, MntC, and capsule polysaccharide were detected by qRT-PCR. Antigen expression was indirectly confirmed by increases in antibody titer during the course of infection from acute to convalescent phase. Demonstration of bacterial transcript expression together with immunological response to the SA4Ag antigens in a clinically relevant patient population provides support for inclusion of these antigens in a prophylactic vaccine.

## Introduction

The Gram positive bacterium *S*. *aureus* is both a human commensal and opportunistic pathogen. The disease syndromes caused by *S*. *aureus* are diverse, ranging from relatively mild skin infections to more severe and invasive infections including endocarditis, necrotizing fasciitis, osteomyelitis, and pneumonia. While *S*. *aureus* is the primary cause of healthcare-associated infection, with nearly 1% of all US hospital inpatient admissions affected by *S*. *aureus* disease, community-associated *S*. *aureus* disease has become prominent both in the US and globally [1]. Clinical treatment options have been impacted by the emergence of drug-resistant organisms and the absence of chemotherapeutic agents with novel mechanism(s) of action. While there are no licensed *S*. *aureus* vaccines, there is a clear medical need for a vaccine to prevent the widespread disease manifestations of *S*. *aureus* and reduce the substantial burden that the pathogen imposes for healthcare systems.

*S*. *aureus* uses several virulence strategies to cause diverse pathologies. An efficacious prophylactic vaccine must generate immune responses that functionally challenge several different virulence factors. A prophylactic *S*. *aureus* 4-antigen (SA4Ag) vaccine comprising two capsular polysaccharide conjugates (CP5-CRM_197_ and CP8-CRM_197_), together with recombinant protein antigens clumping factor A (ClfA) and manganese transporter C (MntC) is currently in development [2]. Capsular polysaccharides (CP) prevent opsonophagocytosis of bacteria, a well-established immune evasion strategy; however, antibodies to CP facilitate phagocytosis by host immune cells and lead to functional antibacterial activity [3]. ClfA is a highly conserved (>88% sequence identity) cell surface adhesin protein detected in >99% of *S*. *aureus* clinical isolates [4]. ClfA-mediated binding of *S*. *aureus* to the C-terminus of the host plasma fibrinogen gamma chain promotes fibrin cross-linking, pathogen binding to platelets and thrombus formation [5]. The fourth component of the SA4Ag vaccine, MntC, is a highly conserved (>98% sequence identity) manganese binding surface lipoprotein [2]. Manganese is an essential cofactor of several *S*. *aureus* enzymes, including superoxide dismutase (SOD). Manganese-dependent bacterial SODs play a key role in immune evasion by detoxifying superoxide metabolites generated by engulfing neutrophils [6].

Understanding the expression of these candidate *S*. *aureus* vaccine antigens during clinical infection is essential. We therefore conducted a prospective observational study of *S*. *aureus* antigen expression and antibody response to these antigens in early stage bacteremia and wound infections in hospitalized patients.

## Results

### Clinical study population

**Fig 1:**
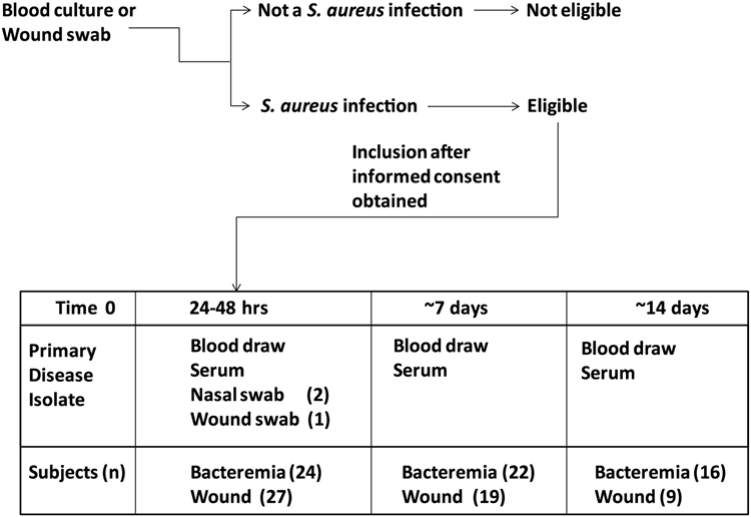
Study design. Although not indicated, additional blood or wound swab samples were recovered from some of the patients over the course of the study.

The overall patient flow is illustrated in Fig. 1. Fifty-one patients were enrolled in the study including 27 with a *S*. *aureus* wound infection and 24 with culture-proven *S*. *aureus* bacteremia. Twenty-five patients completed all study procedures; 16 patients completed only two blood sample time points and ten patients completed only the initial blood sample time point, either because of discharge from the hospital (n = 24) or death (n = 2). Antibiotic therapy was initiated prior to study inclusion in most patients (82%). Clinical characteristics of the study population are summarized in Table 1. *S*. *aureus* nasal carriage was detected in both bacteremic (n = 15) and wound infection (n = 13) patients.

**Table 1:** Clinical characteristics of the study population.

Number enrolled	51
Gender	31 male/20 female
Median age	62 y (range 20–96 y)
Hospital (n)	VU University Medical Center (20)
	Amphia Hospital (31)
Infection type (n)	wound (27)
	blood (24)
Nasal carriage	28 (55%)^a^
History of *S*. *aureus* infection	13 (25%)^a^
Antibiotic treatment before inclusion	42 (82%)^a^
Foreign body associated	13 (25%)^a^
Predisposing condition	24 (47%)^a^

^a^Number of patients with the condition; percent of total patients with the condition is indicated in parentheses.

### Characterization of *S*. *aureus* isolates associated with disease

Invasive *S*. *aureus* isolates were recovered from each patient at the time of enrollment, and serial isolates were obtained from seven wound infection patients and from three bacteremic patients. In addition to the primary invasive isolates, 24 isolates were collected from other sources (e.g., localized infection sites, catheters, pacemaker cords, urine). All isolates were sensitive to methicillin. Approximately half of the patients had infections subsequent to surgical procedures (23/51; seven bacteremia, 16 wound) and these wound infections all occurred at the surgical site. The remainder of patients (28) had infections that were non-surgical in origin and were mostly bacteremic. Descriptive detail for each strain is presented in S1 Table.

**Fig 2:**
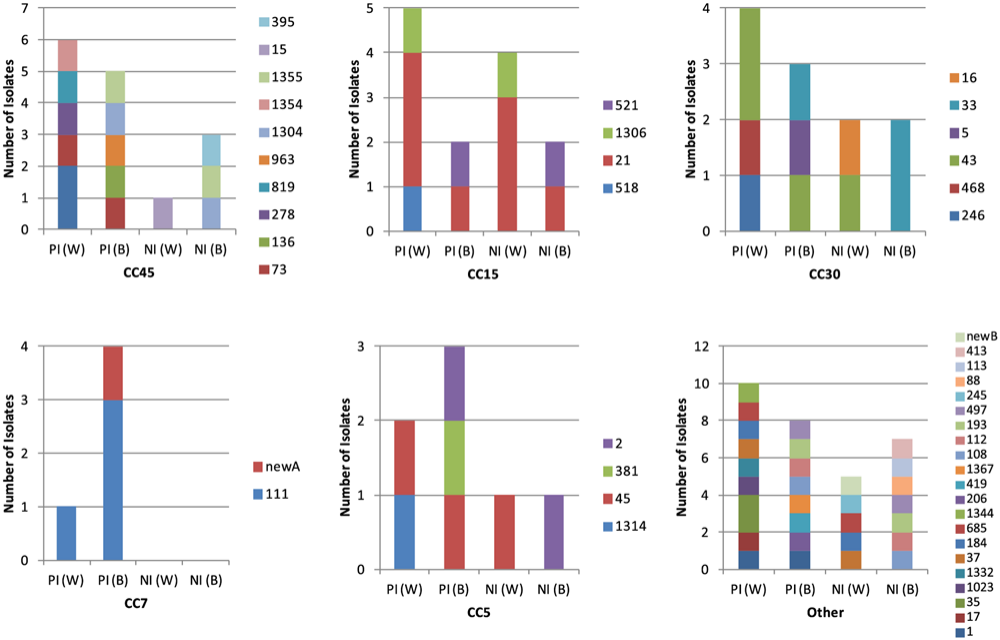
Distribution of isolate *spa* types in clonal complexes (CC). Major CCs are shown (CC45, 15, 30, 7, and 5) together with *spa* types of isolates associated with the respective CCs. Additional *spa* types for the remainder of the isolates are indicated as “Other”. PI (W), primary isolate from wound; PI (B), primary isolate from blood; NI (W), nasal isolate from wound patient; NI (B), nasal isolate from bacteremia patient.

*Spa* typing, which categorizes bacterial relatedness based on the DNA sequence of the protein A gene (*spa*), was conducted to assess the diversity of disease-causing strains in this patient group. Forty different *spa* types were identified among the invasive isolates, illustrating a high degree of heterogeneity (Fig. 2). If patients had multiple isolates from different disease-associated specimens, the isolates had identical *spa* type 86% of the time (6/7) in the wound cohort, and 82% of the time (14/17) in the bacteremic cohort. In four patients the *spa* type of the primary isolate was distinct from the secondary isolate. In two instances the *spa* types of the isolates were slightly different while the multi-locus sequence type (MLST) profiles were the same, indicating that the isolates may be related. For the other two cases, both the MLST and *spa* genotypes were different, indicating that these two strains were not related.

MLST analysis revealed that the *S*. *aureus* disease isolates belonged to the 5 major global clonal complexes (CC) common to The Netherlands (Fig. 2). Wound and bacteremia isolates were distributed evenly within the CCs, with the exception of CC7 (where 4/5 isolates were associated with bacteremia) and CC15 primary disease isolates (where 5/7 isolates were associated with wound infections).

**Fig 3:**
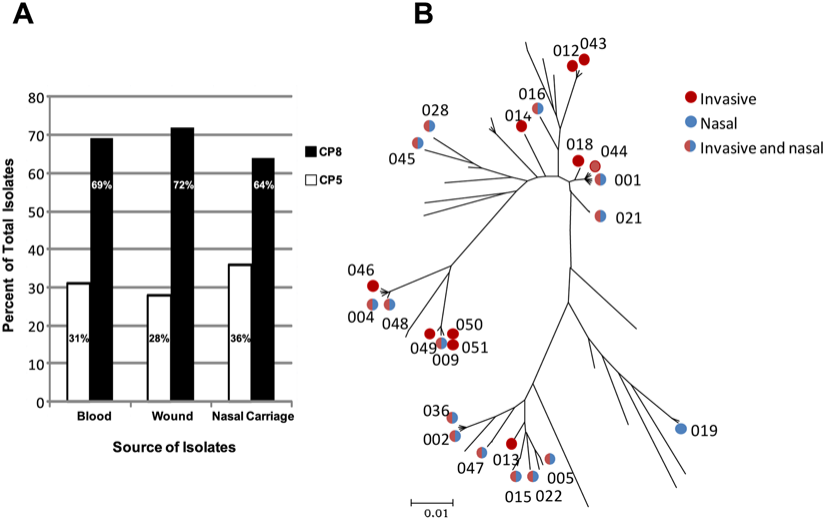
Characterization of isolates by capsule type and *clfA* allele. Panel A. CP5 (open bar) and CP8 (solid bar) distribution in wound, blood, and nasal isolates. Panel B. *clfA* alleles carried by *S*. *aureus* isolates displayed on phylogenetic tree. Alleles occurring only in invasive isolates are indicated by red circles, those detected only in nasal isolates are indicated by blue circles, and those alleles found in both types of isolates are indicated by the blue-red circles.

The capsule polysaccharide (CP) genotype was determined for each isolate by PCR detection of either the *cap5J* or *cap8J* gene. As outlined in Fig. 3A, CP8 was the prevalent capsule genotype in each of the disease-related isolate sub-populations. All disease isolates contained *clfA* and *mntC*. Anderson et al [2] previously demonstrated that MntC is highly conserved (maximum sequence ID 99%), whereas ClfA protein variants are less well conserved [3]. ClfA variant sequences identified in this study were within the maximum sequence diversity known for ClfA (Fig. 3B). Of the over 20 ClfA variants detected, ten were associated with primary disease-causing strains from multiple patients (n = 39). Most of these isolates had different *spa* sequences indicating that the strains were not closely related. Comparison of ClfA variants detected in bacteremic versus wound patients revealed that 11 variants were unique to wound patients, six to bacteremic patients and eight were detected in both cohorts. For isolates other than the primary disease isolate, 14 ClfA variants were observed and in two cases these did not match the variant carried by the primary disease isolate. When comparing ClfA variant and capsule type, it was noted that different isolates carrying the same protein variant were always of the same capsule type.

### The relationship between disease and carriage isolates

Nasal carriage isolates were identified in 28 (55%) of the patients (15 bacteremic and 13 wound patients). As with the disease isolates, the nasal carriage isolates were diverse. In addition to 16 *spa* types shared with the invasive isolates, there were also seven *spa* types unique to nasal isolates (Fig. 2). Clonal complex distribution was very similar to that of the invasive isolates except that there were no CC7 isolates in the carriage subset. Again, CP8 was the predominant capsule type (64%, Fig. 3A). All isolates were positive for *clfA* and *mntC*. There were 15 ClfA variants observed in the carriage isolates, including one (ClfA_019) that was not seen in the disease isolates (Fig. 3B).

*Spa*-matched disease/carriage isolates were detected for 18 (64%) of the 28 carriage-positive patients and these were evenly distributed between both disease cohorts. Also, similar numbers of surgical (11/16) and non-surgical (7/12) infections had matched nasal carriage isolates. When the *spa* types of carriage and disease isolates did not match (10/28 or 36%), there was no discernible difference between the numbers of bacteremic (6/10) versus wound (4/10) patients with heterologous carriage strains. Carriage isolates were identified that matched the *spa* types of primary disease isolates from other patients, highlighting the potential of strains to spread within populations (Fig. 2).

### Gene expression analysis

The RNA expression profile of *S*. *aureus* genes associated with virulence was evaluated in 25 wound swabs (23 patients, two with serial swabs) and 53 nasal swabs (51 patients, two with duplicate swabs). Despite the administration of antibiotics to patients prior to swab sampling, *S*. *aureus* 16S rRNA, a highly abundant transcript, was detected in most of these samples (94% and 87% of the wound and nasal swabs, respectively). Most (27/28) patients with culture-positive nasal swabs also demonstrated 16S rRNA positivity. Some (19/23) patients with culture-negative nasal swabs were positive for 16S rRNA transcripts, illustrating the greater sensitivity of the molecular approach. Samples were tested for *clfA*, *mntC*, *capH*, *isdB*, and for the biofilm-associated *icaB* gene transcripts [7]. There were eight wound and four nasal swabs with detectable virulence gene transcripts, primarily *mntC* and *clfA; icaB* was not detected from any swab. The *S*. *aureus* triosephosphate isomerase (*tpi*) gene was included as a control for expression from a single-copy housekeeping gene. Of the twelve swabs, 16S rRNA and *tpi* transcripts were both detected in only five wound and three nasal swabs. The relative copy number of the 16S rRNA, *tpi* and the respective virulence gene transcripts are indicated in Table 2. The wound samples had higher *tpi* expression levels than the nasal swabs, indicating a higher bacterial load. In both wound and nasal swabs, relative transcript copy numbers demonstrate that *mntC* expression levels were typically higher than those of *clfA*. Expression of *capH* and *isdB* was detected in two wound samples but not in the nasal swabs. In both of these wound samples (patients 100106 and 100123), *cap* transcripts were specific for CP8 and consistent with the respective CP genotype of the invasive isolates recovered from these patients.

**Table 2:** *In vivo* expression of *S*. *aureus* genes in humans with confirmed *S*. *aureus* infection.

*S*. *aureus* RNA Expression (Relative Copy Number)^a^								
Patient	Swab Type	16S rRNA	*Tpi*	*clfA*	*mntC*	*capH* (type)	*isdB*	*IcaB*
100106	Wound (diabetic foot ulcer)	1.4 × 10^7^	1081	1455	3723	118 (8)	49	-
100123	Wound (abdominal aneurism)	6.8 × 10^6^	1017	1533	14642	1117 (8)	2841	-
100130	Wound (knee bursa)	1.9 × 10^5^	72	203	283	-	-	-
100131	Wound (hip, surgical)	7.2 × 10^5^	396	386	3735	-	-	-
100138	Wound (thoracic drain)	3.5 × 10^5^	42	21	359	-	-	-
100108	Nasal	7.9 × 10^5^	221	585	3039	-	-	-
100127	Nasal	1.1 × 10^5^	32	33	156	-	-	-
100152	Nasal	3.8 × 10^4^	20	66	119	-	-	-

^a^Total RNA was extracted from wound and nasal carriage swabs. Following RT-PCR, RNA copy number for several *S*. *aureus* gene targets was calculated using gene-specific standard curves. qRT-PCR assay limit of detection (LOD) is 20 copies and samples below the LOD are marked as (-). Targeted genes include *tpi* (triosephosphate isomerase), *clfA* (clumping factor A), *mntC* (manganese transporter C), *capH* (capsule H), *isdB* (iron-regulated surface determinant B), and *icaB* (intercellular adhesion B).

### Serological evaluation

Patients were enrolled in this study only after confirmation of a *S*. *aureus* infection. As a consequence, there were no pre-infection sera available for comparative analysis. The serum geometric mean antibody titer (GMT) values determined using the competitive Luminex immunoassay (cLIA) are summarized in Table 3. Antibody titer specific for each of the four candidate vaccine antigens was detected in this study population. Comparisons of GMT values for the four antigens between patients with wound and blood infections are outlined in Table 3. Unlike the CP antigens, there was a trend for higher serum antibody titer for both protein antigens in bacteremic than in wound patients.

**Table 3:** Serum Geometric Mean Titer (GMT) values from Competitive Luminex Immunoassay (cLIA)^a^.

Antigen		All Patients		Wound	Blood	p Value^b^		CP5 Infected	CP8 Infected	p Value^b^
		(n = 51)	(n = 27)	(n = 24)			(n = 14)	(n = 36)		
		GMT		GMT	GMT			GMT	GMT	
		(95% CI)		(95% CI)	(95% CI)			(95% CI)	(95% CI)	
CP5		143.63		148.20	138.68			260.90	115.02	0.043*
		(100.0, 206.01)		(98.65, 222.56)	(72.32, 265.95)	0.860		(121.1, 562.11)	(76.04, 173.99)	
CP8		120.15		145.40	96.64			85.18	138.76	0.215
		(85.06, 169.73)		(86.25, 245.21)	(60.85, 154.43)	0.243		(45.97, 157.84)	(89.75, 214.53)	
ClfA		38.60		36.50	41.11			27.76	43.42	0.032
		(30.39, 49.03)		(26.87, 49.59)	(27.64, 61.16)	0.623		(21.10, 36.54)	(31.57, 59.71)	
MntC		105.24		80.20	144.84			127.33	94.07	0.521
		(69.38, 159.63)		(45.35, 141.71)	(76.95, 272.64)	0.157		(49.15, 329.86)	(57.99, 152.62)	

^a^A qualified multiplex 3+1 competitive Luminex immunoassay (cLIA) was used to measure serum antibody titers specific for ClfA, MntC, and capsule polysaccharide types 5 and 8. For calculating GMT when titers were below the limit of quantitation (BLQ), a value of ½ LLOQ for each assay was used: ½ LLOQ for ClfA = 24.45; for MntC = 21.35; for CP5 = 23.5; and for CP8 = 18.3 U/mL. GMT calculated using highest titer observed for each patient.

^b^Wilcoxon test or 2 sample t-test.

* = statistical significance; CI = Confidence Interval; GMT = geometric mean antibody titer.

**Fig 4:**
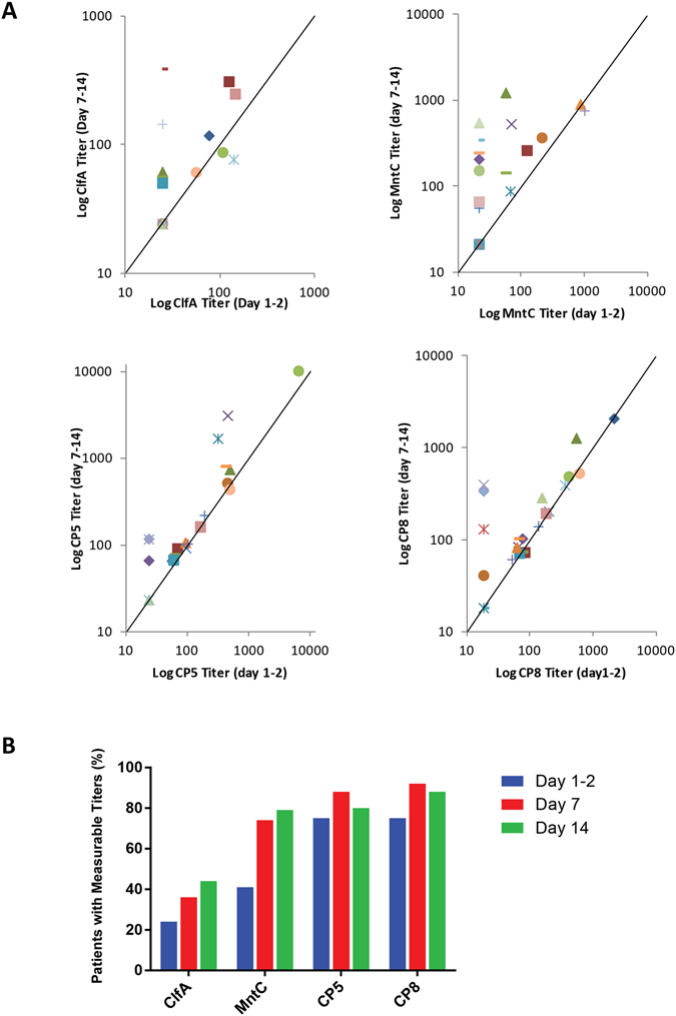
Antibody titers to the SA4Ag vaccine antigens. Panel A. Antibody titers to the four antigens at study baseline (day 1–2) to Day 7–14. Scatter plots of titers are shown for the 25 patients who had blood samples drawn at three different times over the two-week course of the study. If the titer was reported as “below limit of quantitation”, a value that is ½ LLOQ was used in the plots; for ClfA, MntC, CP5 and CP8, the values are 24.45, 21.35, 23.5 and 18.3 U/mL, respectively. Points above the diagonal line indicate a higher titer in the later samples. Panel B. Percentage of patients with titers above the limit of quantitation over time. Patient population is as described for Panel A (above).

Most of the patients who were enrolled were infected with CP8 isolates (Fig. 3A). A comparison of serum antibody titer to the four antigens between patients with CP5 and CP8 infections is also captured in Table 3. In patients with CP8 invasive isolates, the CP8 GMT was greater than that of CP5, although the difference was not significant (p = 0.215). Antibody titers to CP5 were higher than to CP8 in patients with CP5 infections, which reached significance (p = 0.043). Serum titers to the SA4Ag vaccine antigens were monitored from the acute to the convalescent phase of the infection. The data in Fig. 4A illustrate that the antibody titers tended to rise from the acute phase of infection (day 1–2) to the convalescent phase (day 14). In addition, the percentage of patients with serum titers above the assay limit of quantitation for CP8, ClfA, and MntC increased over that 2-week interval (Fig. 4B).

## Discussion

In the current study a cohort of 51 patients ≥ 18 years of age with diagnosed *S*. *aureus* wound or bloodstream infections were enrolled at two hospitals in The Netherlands. Invasive and carriage isolates from these patients were genotypically characterized. Nasal carriage of *S*. *aureus* is considered a risk factor for infection in certain patient populations [8] [9] [10,11] [12]. In this study, a nasal carriage isolate was recovered in 55% of the patients. In those patients where a carriage isolate was obtained, the invasive isolate was genotypically indistinguishable in 69% (11/16) of surgical patients and 58% (7/12) of non-surgical patients. Overall, nasal carriage isolates were linked to disease strains in approximately 35% of patients. All isolates, regardless of origin, were sensitive to methicillin. This corresponds well with the observed low incidence of methicillin resistant *S*. *aureus* (MRSA) isolates in The Netherlands and points to the effectiveness of the “search and destroy” strategy to control MRSA [13]. Although the isolates in this study were methicillin sensitive, two bacteremic patients (100108 and 100126) died from sepsis, highlighting the fact that MSSA strains continue to be significant pathogens.

Despite the size of the study population and the geographic proximity of the two study sites, there was considerable genotypic heterogeneity in the *S*. *aureus* disease isolates. Comparative analysis of the nasal carriage (PFESA317) and primary disease isolate (PFESA304) from patient 100125 illustrates the discriminatory power of *spa* typing. The isolates have identical capsule, *clfA* and MLST genotypes, but their *spa* profiles differ from each other by a small insertion and sequence replacement in two regions of the locus (S1 Table). Examples of isolates with *spa* identity but distinct *clfA* and MLST alleles were also identified. Of the nine *spa* 21 type isolates characterized, four had *clfA* 015–1 and ST15 genotypes while five had *clfA*047–1 and ST582 genotypes (S1 Table). Bacterial genotyping using whole genome sequence data will provide the best discrimination between closely related isolates [14].

In other studies of invasive *S*. *aureus* disease in Europe, the major CCs observed are CC5, 8, 15, 30 and 45 [15] [16]. The major CCs were also well represented in the current study, but there were also several CC7 isolates (n = 14). Other studies of *S*. *aureus* bacteremic infections in The Netherlands have recovered CC7 strains, so the current findings correlate well with previous results. However, in contrast to the current study, others have reported that CC7 isolates are also recovered from nasal carriage [17]. Except for a small number of CC6, CC34, CC72, and CC121 isolates, each CC in this patient population is represented in the larger collection of *S*. *aureus* isolates reported earlier [4].

The second objective of this study was to determine whether SA4Ag vaccine antigens are expressed during infection or carriage. Two approaches were used to detect antigen expression. The first was qRT-PCR and the second was a serological assay. For a vaccine strategy to be effective, the vaccine must contain antigens expressed by *S*. *aureus* during the course of natural infection. Many studies use PCR to detect the presence of specific virulence genes in bacteria isolated from infections as a measure of their pathogenic potential [16] [18]. In recent studies with human subjects, *S*. *aureus* RNA transcript levels were quantitated using qRT-PCR in persistent, highly-colonized nasal carriers [19]. Similarly *S*. *aureus* transcript levels were monitored in subjects with cutaneous abscesses using microarray hybridization and qRT-PCR [20]. In the current study, gene expression of virulence factors was measured in different types of human infections and in nasal carriage. This is the first study to assess antigen expression by diverse *S*. *aureus* strains during infection. The nature of the study presented substantial technical challenges. For example, wound and nasal swabs were collected only following confirmation of a *S*. *aureus* infection in patients. Even prior to confirmation, the standard of care is to administer antibiotics. Consequently, patients in the study may have been on antibiotic therapy for up to 48 hours before the wound/nasal swabs were obtained. This could negatively impact recovery of bacteria on swabs for antigen expression analysis, and low numbers of bacteria in tissue samples has been identified as a limitation in this type of experiment [20]. The frequency of *tpi* negative samples points to a less than optimal recovery of bacteria and/or bacterial RNA from many of the swabs. Despite the experimental challenges, RNA transcripts corresponding to capsule, *clfA* and *mntC* genes were observed, with consistent detection of *clfA* and *mntC* transcripts where expression of *tpi* was also detected. Burian et al [19] also reported that *clfA* transcripts were seen in nasal samples from colonized subjects. In our study, *mntC* was always expressed at higher levels than other genes in nose and wound samples. Date et al. [20] also observed that *mntC* expression is slightly up-regulated in the cutaneous abscess study. Previous studies [21] [22] demonstrated a temporal expression of *S*. *aureus* virulence factors in preclinical in vivo studies. Given that gene expression was examined at only one time point in the current study, the lack of consistent expression of other virulence factor genes is not surprising. Also, transcript stability may differ between these genes, especially in the polycistronic capsule operon, making it more difficult to monitor expression with this approach.

An alternative approach to monitor antigen expression indirectly is to measure the presence of bacterial antigen-specific antibodies in serum [23] [24], [25], [26] [27]. The lack of serum samples from these patients prior to infection for comparative analysis is one limitation of the current study. However, serology results demonstrated that most of the patients responded to ClfA and MntC, and the percentage of responders increased over time. Antibodies are generated as a result of immune exposure to the organism and the detection of specific antibodies indicates that the subject has encountered the *S*. *aureus* antigen. The quality of native immune responses was illustrated by Hawkins et al [28] who mimicked the natural situation by immunizing non-human primates with killed *S*. *aureus* cells. These animals generated antibodies that bound to ClfA but did not inhibit the binding of ClfA to fibrinogen, its natural ligand. Importantly, the cLIA titers to ClfA observed following natural infection in the current study are several orders of magnitude less than titers observed in subjects receiving the investigational SA4Ag vaccine [29].

Patients in the current study with documented *S*. *aureus* infections also had measurable and specific antibody titer to CP5 and CP8. Specific antibody titers to the respective CP antigens are greater in patients infected with *S*. *aureus* isolates expressing the same CP type. Despite the challenges described earlier, expression of *capH* was detected in two wound swabs and in both cases *cap* transcripts were specific for CP8, consistent with the capsule genotype identified in the invasive isolate.

*S*. *aureus* employs a number of strategies to enable both colonization of the host and survival *in vivo*. The bacterium uses relevant virulence factors, including capsule polysaccharide, binding proteins and toxins, to adapt to a variety of host niches and cause a multitude of diverse infections. The broad spectrum of pathogenic mechanisms renders *S*. *aureus* a challenging vaccine target. An essential characteristic for a *S*. *aureus* vaccine antigen is that immunization must elicit a functional response capable of killing the diverse collection of disease-causing isolates. Thus, the vaccine antigen must be (i) present in the genome of geographically dispersed invasive isolates, (ii) highly conserved, (iii) expressed by the pathogen in host micro-environments, and (iv) accessible to antibodies. In this study we demonstrated that the four antigens present in the investigational SA4Ag vaccine were distributed across disease and carriage isolates. We obtained evidence, both direct (from wound) and indirect (from sera), that these antigens are expressed during disease, providing assurance that they will be accessible to antibodies generated by the SA4Ag vaccine in patients who are at risk of invasive *S*. *aureus* disease.

## Materials and Methods

### Patients and study design

Between March 2008 and March 2009 patients were recruited from two hospitals, a tertiary university hospital (VU University Medical Center in Amsterdam, The Netherlands) and a large teaching hospital (Amphia Hospital in Breda, The Netherlands). The medical ethical committee of each study site approved the study. Adult (≥18 years) hospitalized patients with culture proven *S*. *aureus* wound infections or bacteremia, were assessed for eligibility. Definitive microbiological diagnosis was required within 48 hours after cultures were obtained. Patients admitted to the intensive care unit, those with hematological diseases or being treated with immunosuppressive drugs were excluded. Patients were included after written informed consent had been given.

### Sample collection

At inclusion into the study, blood was drawn and the anterior nares were swabbed with duplicate swabs. One swab was cultured on blood agar by standard microbiological methods and the second was frozen at -80°C for real-time, quantitative, reverse-transcriptase PCR (qRT-PCR) analysis. Culture isolates obtained from blood and/or wound sites were handled similarly, as were isolates obtained from any other accessible infection site(s) when present. *S*. *aureus* identification was confirmed by coagulase and/or PCR testing; methicillin susceptibility was assessed by standard antibiotic susceptibility testing. Blood samples were also drawn at one and two weeks after the initial assessment to evaluate immunological changes over time.

### Molecular analysis of *S*. *aureus* isolates

*S*. *aureus* isolates collected at time of diagnosis (primary disease isolates), subsequently isolated disease isolates and nasal isolates were characterized by MLST (according to protocols at saureus.mlst.net/[30]), *spa* typing [31] [32], capsule serotype-specific PCR [4], and *clfA* and *mntC* DNA sequencing [2][4]. Genomic DNA was isolated from *S*. *aureus* cultures grown in tryptic soy broth (TSB, Difco) using the Qiagen DNeasy blood and tissue kit according to manufacturer’s instructions, except for the inclusion of lysostaphin (25–30U per 200 μL) in the first step. *Spa* typing was performed using the *spa* DNA sequence information and the eGenomics software (http://epigene.egenomics.com). ClfA protein variants were assigned as previously reported [4]. For bioinformatics analysis, multiple alignments were created with MAFFT [33]. Phylogenetic trees were constructed with MEGA 4[34].

### Gene expression analysis

Wound and nasal carriage swabs were examined for gene expression by qRT-PCR. Swabs were considered of sufficient quality for qRT-PCR if they were positive using 16S rRNA and *tpi* primer/probe sets. *In vivo* antigen expression was monitored for *clfA*, *capH*, *mntC*, iron surface binding protein B (*isdB*), and intercellular adhesion protein B (*icaB*). RNA was extracted from bacteria on the swabs using the RiboPure kit (Ambion) and converted to cDNA using the Quantitect kit (Qiagen) as recommended by the manufacturers. qRT-PCR reactions were conducted on the 7500 Real Time PCR System (Applied Biosystems). The analysis performed by the 7500 System SDS Software v1.3.1 generated the threshold cycle (Ct) for each sample. The relative transcript copy number was determined from corresponding standard curves generated using 10^2^ to 10^7^ copies of *S*. *aureus* genomic DNA.

### Competitive Luminex Immunoassay (cLIA)

A qualified multiplex cLIA was developed to indirectly measure the level of functional serum antibody titer to each of the four SA4Ag vaccine antigens. Each antigen was coupled to a distinct fluorescent Luminex microsphere. Antigen-coated microspheres were incubated overnight at 4°C with appropriately diluted serum samples, controls, or reference standard serum. Antigen-specific functional mouse monoclonal antibodies (mAbs) were then added to the microsphere/serum mixture and bound mAbs were detected with R-Phycoerythrin (PE) labeled rat anti-mouse IgG1 secondary antibody (Southern Biotech) using a BioPlex reader (BioRad) to measure fluorescence. As this is a competitive assay, the magnitude of the fluorescent PE signal is inversely proportional to the amount of antigen-specific antibody in the sample.

### Statistical analysis

The cLIA serology data were descriptively summarized with GMT and corresponding 95% confidence intervals (CI). If a titer was below the limit of quantitation (LLOQ), ½ × lower limit of quantitation (½ LLOQ) was used to impute assay values when calculating GMT.

The CIs were constructed by back transformation of the confidence limits computed for the mean of the logarithmically transformed assay data based on Student t distribution. P-values based on 2-sample t-test or Wilcoxon test were presented to identify potential differences in immunoglobulin concentration.

## Supporting Information

S1 Table Characterization of *S*. *aureus* Isolates. (PDF)

S2 Table qRT-PCR Results for Nasal and Wound Samples. (PDF)
