# Supplementary material for: Evaluation of Approaches to Monitor Staphylococcus aureus Virulence Factor Expression during Human Disease
Source: PLoS One. 2015 Feb 26;10(2):e0116945. doi: 10.1371/journal.pone.0116945 (PMC4342157; doi:10.1371/journal.pone.0116945)
Supplement: S1 Table — (PDF) [file pone.0116945.s001.pdf]

Table S1. Characterization of *S. aureus* Isolates

Alias	Invasive/ Carriage[a]	Site Id[b]	Patient	Age	Gender	Culture Source	*clfA* -N	*mntC* - N	Capsule Type	Spa Genotype	Spa Type	Clonal Complex
PFESA0369	Invasive*	NLD1	100101	29	Male	Wound (surgical back)	ClfA_015-1	mntC-001-1	8	UJGBBGGJAGJ	21	CC15
PFESA0324	Carriage	NLD1	100101	29	Male	Nasal	ClfA_015-1	mntC-001-1	8	UJGBBGGJAGJ	21	CC15
PFESA0389	Invasive*	NLD2	100102	43	Female	Wound (leg)	ClfA_012-1	mntC-001-1	8	UPE	1023	CC1
PFESA0360	Invasive	NLD2	100102	43	Female	Wound (leg)	ClfA_012-1	mntC-001-1	8	UPE	1023	CC1
PFESA0364	Invasive*	NLD1	100103	33	Female	Wound (panaritium)	ClfA_015-1	mntC-001-1	8	I2GBBAGJ	1306	CC15
PFESA0355	Carriage	NLD1	100103	33	Female	Nasal	ClfA_015-1	mntC-001-1	8	I2GBBAGJ	1306	CC15
PFESA0325	Carriage	NLD2	100104	49	Male	Nasal	ClfA_028-1	mntC-001-8	8	I2Z2EGMMMMJH2	685	CC121
PFESA0299	Carriage	NLD2	100104	49	Male	Nasal	ClfA_028-1	mntC-001-8	8	I2Z2EGMMMMJH2	685	CC121
PFESA0405	Invasive*	NLD2	100104	49	Male	Wound (knee)	ClfA_028-1	mntC-001-8	8	I2Z2EGMMMMJH2	685	CC121
PFESA0379	Invasive*	NLD1	100105	41	Male	Wound (bone skin graft)	ClfA_012-1	mntC-001-1	8	UJFKBPE	35	CC1
PFESA0382	Invasive*	NLD2	100106	38	Male	Wound (foot)	ClfA_015-1	mntC-001-1	8	UJGBBBGGJAGJ	518	CC15
PFESA0390	Invasive	NLD2	100106	38	Male	Wound (foot #2)	ClfA_015-1	mntC-001-1	8	UJGBBBGGJAGJ	518	CC15
PFESA0380	Invasive*	NLD1	100107	45	Male	Wound (hip)	ClfA_050-1	mntC-006-1	8	A2AKBEMBKB	42	CC45
PFESA0356	Carriage	NLD1	100107	45	Male	Nasal	ClfA_004-1	mntC-001-7	8	WGKAKAOMQQQ	16	CC30
PFESA0306	Invasive**	NLD2	100108	83	Male	Blood	ClfA_005-1	mntC-001-1	5	ZFGU2DMGGGGM	1367	CC25
PFESA0291	Invasive**	NLD1	100109	22	Female	Blood	ClfA_004-1	mntC-001-7	8	WGKAKAOMQ	43	CC30
PFESA0377	Invasive*	NLD2	100110	45	Male	Wound (hip)	ClfA_046-1	mntC-001-7	8	WGKAKAOMQ	43	CC30
PFESA0352	Carriage	NLD2	100110	45	Male	Nasal	ClfA_046-1	mntC-001-7	8	WGKAKAOMQ	43	CC30
PFESA0311	Invasive**	NLD1	100111	67	Female	Blood	ClfA_009-1	mntC-006-1	8	XKAKBEMBKB	73	CC45
PFESA0292	Carriage	NLD1	100111	67	Female	Nasal	ClfA_001-1	mntC-001-13	5	UJGFMBPB	413	CC97
PFESA0298	Invasive**	NLD2	100112	70	Male	Blood	ClfA_036-1	mntC-001-5	5	TJMBMDMGMK	2	CC5
PFESA0391	Invasive	NLD2	100112	70	Male	Heart valve	ClfA_036-1	mntC-001-5	5	TJMBMDMGMK	2	CC5
PFESA0326	Carriage	NLD2	100112	70	Male	Nasal	ClfA_036-1	mntC-001-5	5	TJMBMDMGMK	2	CC5
PFESA0404	Invasive	NLD2	100112	70	Male	Urine	ClfA_036-1	mntC-001-5	5	TJMBMDMGMK	2	CC5
PFESA0312	Carriage	NLD1	100113	69	Male	Nasal	ClfA_016-1	mntC-001-6	5	UJGFGMDMGGM	193	CC72
PFESA0301	Invasive**	NLD1	100113	69	Male	Blood	ClfA_016-1	mntC-001-6	5	UJGFGMDMGGM	193	CD72
PFESA0327	Invasive	NLD2	100114	71	Male	CVD catheter	ClfA_022-1	mntC-006-2	5	TJNCMOR	112	CC22
PFESA0328	Carriage	NLD2	100114	71	Male	Nasal	ClfA_022-1	mntC-006-2	5	TJNCMOR	112	CC22
PFESA0329	Invasive	NLD2	100114	71	Male	Catheter entrance (arm)	ClfA_022-1	mntC-006-2	5	TJNCMOR	112	CC22
PFESA0308	Invasive**	NLD2	100114	71	Male	Blood	ClfA_022-1	mntC-006-2	5	TJNCMOR	112	CC22
PFESA0381	Invasive*	NLD1	100115	52	Male	Wound (surgical-chest)	ClfA_002-1	mntC-001-5	5	TJAMGMK	1314	CC5
PFESA0385	Invasive	NLD1	100117	54	Male	Wound (phlegmone hand)	ClfA_047-1	mntC-001-1	8	UJGBBGGJAGJ	21	CC15
PFESA0313	Carriage	NLD1	100117	54	Male	Nasal	ClfA_047-1	mntC-001-1	8	UJGBBGGJAGJ	21	CC15
PFESA0302	Invasive**	NLD1	100117	54	Male	Blood	ClfA_047-1	mntC-001-1	8	UJGBBGGJAGJ	21	CC15
PFESA0392	Invasive*	NLD2	100118	62	Female	Wound (ankle)	ClfA_018-1	mntC-001-1	8	UJFMBGJAGJ	111	CC7
PFESA0332	Carriage	NLD2	100118	62	Female	Nasal	ClfA_001-1	mntC-001-1	5	YHFMBQBLO	245	CC8
PFESA0386	Invasive	NLD1	100119	74	Male	Wound	ClfA_051-1	mntC-006-1	8	A2AKBBMBKB	136	CC45

PFESA0303	Invasive**	NLD1	100119	74	Male	Blood	ClfA_051-1	mntC-006-1	8	A2AKBBMBKB	136	CC45
PFESA0333	Invasive	NLD2	100120	45	Male	Blood (2nd episode)	ClfA_004-1	mntC-001-7	8	WGKAKAOMQQ	33	CC30
PFESA0338	Carriage	NLD2	100120	45	Male	Nasal	ClfA_004-1	mntC-001-7	8	WGKAKAOMQQ	33	CC30
PFESA0309	Invasive**	NLD2	100120	45	Male	Blood (1st episode)	ClfA_004-1	mntC-001-7	8	WGKAKAOMQQ	33	CC30
PFESA0387	Invasive	NLD1	100121	29	Female	Wound	ClfA_004-1	mntC-001-7	8	ZZ2PNGKBKG	497	CC30/34
PFESA0314	Invasive	NLD1	100121	29	Female	Catheter tip	ClfA_004-1	mntC-001-7	8	ZZ2PNGKBKG	497	CC30/34
PFESA0315	Carriage	NLD1	100121	29	Female	Nasal	ClfA_004-1	mntC-001-7	8	ZZ2PNGKBKG	497	CC30/34
PFESA0293	Invasive**	NLD1	100121	29	Female	Blood	ClfA_004-1	mntC-001-7	8	ZZ2PNGKBKG	497	CC30/34
PFESA0375	Invasive*	NLD2	100122	62	Male	Wound (groin)	ClfA_001-1	mntC-001-1	5	YHGFMBQBLO	1	CC8
PFESA0316	Carriage	NLD1	100123	87	Female	Nasal	ClfA_015-1	mntC-001-1	8	UJGBBGGJAGJ	21	CC15
PFESA0365	Invasive*	NLD1	100123	87	Female	Wound (groin)	ClfA_015-1	mntC-001-1	8	UJGBBGGJAGJ	21	CC15
PFESA0363	Invasive*	NLD2	100124	78	Female	Wound (wrist/arm)	ClfA_009-1	mntC-006-1	8	XKAKBEMBKB	73	CC45
PFESA0317	Carriage	NLD1	100125	22	Male	Nasal	ClfA_009-1	mntC-006-1	8	XKBEMBKB	395	CC45
PFESA0320	Invasive	NLD1	100125	22	Male	Abcess	ClfA_002-1	mntC-001-5	5	TJMBMDMGMK	2	CC5
PFESA0304	Invasive**	NLD1	100125	22	Male	Blood	ClfA_009-1	mntC-006-1	8	XKAKBEMBKE	963	CC45
PFESA0290	Carriage	NLD2	100126	96	Female	Nasal	ClfA_045-1	mntC-001-10	8	ZDMJDM	108	CC50
PFESA0396	Invasive	NLD2	100126	96	Female	Wound (toe-old infection)	ClfA_045-1	mntC-001-10	8	ZDMJDM	108	CC50
PFESA0353	Invasive**	NLD2	100126	96	Female	Blood	ClfA_045-1	mntC-001-10	8	ZDMJDM	108	CC50
PFESA0321	Carriage	NLD1	100127	64	Female	Nasal	ClfA_021-1	mntC-001-1	5	UKGJB	37	CC9
PFESA0366	Invasive*	NLD1	100127	64	Female	Wound (tracheostomia)	ClfA_021-1	mntC-001-1	5	UKGJB	37	CC9
PFESA0300	Invasive**	NLD2	100128	45	Male	Blood	ClfA_002-1	mntC-001-5	5	TK	381	CC5
PFESA0406	Invasive	NLD2	100128	45	Male	Wound (sternum)	ClfA_002-1	mntC-001-5	5	TK	381	CC5
PFESA0319	Carriage	NLD1	100129	83	Female	Nasal	ClfA_022-1	mntC-006-2	5	TJEJNCMOMOKR	113	CC22
PFESA0294	Invasive**	NLD1	100129	83	Female	Blood	ClfA_018-1	mntC-001-1	8	UJFMBGJAGJ	111	CC7
PFESA0378	Invasive*	NLD2	100130	20	Male	Wound (knee)	ClfA_009-1	mntC-006-1	8	XKAKBEEKB	1354	CC45
PFESA0399	Invasive	NLD2	100130	20	Male	Wound (knee)	ClfA_009-1	mntC-006-1	8	XKAKBEEKB	1354	CC45
PFESA0318	Carriage	NLD1	100131	83	Male	Nasal	ClfA_009-1	mntC-006-1	8	A2AKEEMBKB	15	CC45
PFESA0383	Invasive*	NLD1	100131	83	Male	Wound (surgical - hip)	ClfA_013-1	mntC-001-1	5	UR5MFBBLB	1344	CC20
PFESA0334	Invasive**	NLD2	100132	57	Female	Blood	ClfA_018-1	mntC-001-1	8	UJFMBGJAGJ	111	CC7
PFESA0335	Invasive	NLD2	100132	57	Female	Blood (3rd sample)	ClfA_018-1	mntC-001-1	8	UJFMBGJAGJ	111	CC7
PFESA0336	Invasive	NLD2	100132	57	Female	Blood (2nd sample)	ClfA_018-1	mntC-001-1	8	UJFMBGJAGJ	111	CC7
PFESA0397	Invasive	NLD2	100132	57	Female	Wound (back 2nd sample)	ClfA_018-1	mntC-001-1	8	UJFMBGJAGJ	111	CC7
PFESA0368	Invasive	NLD2	100132	57	Female	Wound (back)	ClfA_018-1	mntC-001-1	8	UJFMBGJAGJ	111	CC7
PFESA0388	Invasive*	NLD1	100133	60	Male	Wound	ClfA_047-1	mntC-001-1	8	UJGBBGGJAGJ	21	CC15
PFESA0305	Carriage	NLD1	100133	60	Male	Nasal	ClfA_047-1	mntC-001-1	8	UJGBBGGJAGJ	21	CC15
PFESA0370	Invasive**	NLD2	100134	71	Male	Blood	ClfA_009-1	mntC-006-1	8	X	419	CC45
PFESA0367	Invasive*	NLD1	100135	63	Female	Wound (opening site of IV drip)	ClfA_048-1	mntC-001-7	8	WGAKAOMQ	246	CC30
PFESA0371	Invasive*	NLD2	100136	35	Male	Wound (thorax drain)	ClfA_014-1	mntC-001-1	8	ZDMDMNKB	17	CC59
PFESA0322	Carriage	NLD1	100137	54	Male	Nasal	ClfA_005-1	mntC-001-1	5	ZFGU2DMGGM	184	CC25
PFESA0384	Invasive*	NLD1	100137	54	Male	Wound (traumatic skin defect-back)	ClfA_005-1	mntC-001-1	5	ZFGU2DMGGM	184	CC25

PFESA0372	Invasive*	NLD2	100138	61	Male	Wound (thorax drain)	ClfA_049-1	mntC-006-1	8	A2AKBEMBKB	42	CC45
PFESA0393	Invasive	NLD2	100138	61	Male	Wound (thorax drain 2nd sample)	ClfA_049-1	mntC-006-1	8	A2AKBB	819	CC45
PFESA0323	Carriage	NLD1	100139	78	Male	Nasal	ClfA_009-1	mntC-006-1	8	XKAKBEMEKB	1355	CC45
PFESA0307	Invasive**	NLD1	100139	78	Male	Blood	ClfA_009-1	mntC-006-1	8	XKAKBEMEKB	1355	CC45
PFESA0373	Invasive	NLD2	100140	72	Male	Wound (pacemaker pocket)	ClfA_018-1	mntC-001-1	8	UJFMBGJAGJ	111	CC7
PFESA0337	Invasive**	NLD2	100140	72	Male	Blood	ClfA_018-1	mntC-001-1	8	UJFMBGJAGJ	111	CC7
PFESA0400	Invasive	NLD2	100140	72	Male	Wound (pacemaker pocket)	ClfA_018-1	mntC-001-1	8	UJFMBGJAGJ	111	CC7
PFESA0351	Invasive	NLD2	100140	72	Male	Pacemaker cords	ClfA_018-1	mntC-001-1	8	UJFMBGJAGJ	111	CC7
PFESA0401	Invasive	NLD2	100140	72	Male	Wound (pacemaker pocket tissue)	ClfA_018-1	mntC-001-1	8	UJFMBGJAGJ	111	CC7
PFESA0374	Invasive*	NLD2	100142	43	Male	Wound (bursa)	ClfA_004-1	mntC-001-7	8	WGKAKAOMQ	43	CC30
PFESA0394	Invasive	NLD2	100142	43	Male	Wound (knee)	ClfA_004-1	mntC-001-7	8	WGKAKAOMQ	43	CC30
PFESA0339	Carriage	NLD2	100142	43	Male	Nasal	ClfA_019-1	mntC-001-1	8	YC2BQBLOO	new	CC6
PFESA0357	Invasive*	NLD2	100144	59	Female	Wound (throat)	ClfA_021-1	mntC-001-1	5	UJFAABB	1332	CC9
PFESA0358	Invasive*	NLD2	100146	84	Female	Wound (knee)	ClfA_002-1	mntC-001-5	5	TJMBDMGMK	45	CC5
PFESA0395	Invasive	NLD2	100146	84	Female	Wound (synovium)	ClfA_002-1	mntC-001-5	5	TJMBDMGMK	45	CC5
PFESA0340	Carriage	NLD2	100146	84	Female	Nasal	ClfA_002-1	mntC-001-5	5	TJMBDMGMK	45	CC5
PFESA0359	Invasive*	NLD2	100148	80	Female	Wound (arm)	ClfA_004-1	mntC-001-7	8	XKAKAOMQQ	468	CC30
PFESA0295	Invasive**	NLD2	100150	51	Male	Blood	ClfA_014-1	mntC-001-1	8	ZDMNKB	206	CC59
PFESA0343	Invasive	NLD2	100150	51	Male	Sternum puncture	ClfA_014-1	mntC-001-1	8	ZDMNKB	206	CC59
PFESA0296	Invasive**	NLD2	100152	68	Male	Blood	ClfA_015-1	mntC-001-1	8	UJGEBGGJAGJ	521	CC15
PFESA0341	Invasive	NLD2	100152	68	Male	Ankle punctate	ClfA_015-1	mntC-001-1	8	UJGEBGGJAGJ	521	CC15
PFESA0342	Invasive	NLD2	100152	68	Male	Elbow punctate	ClfA_015-1	mntC-001-1	8	UJGEBGGJAGJ	521	CC15
PFESA0344	Carriage	NLD2	100152	68	Male	Nasal	ClfA_015-1	mntC-001-1	8	UJGEBGGJAGJ	521	CC15
PFESA0376	Invasive	NLD2	100154	64	Male	Wound (foot)	ClfA_018-1	mntC-001-1	8	UJFAABB	1332	CC7
PFESA0354	Invasive**	NLD2	100154	64	Male	Blood	ClfA_018-1	mntC-001-1	8	UJFMBGJAAGJ	new	CC7
PFESA0361	Invasive*	NLD2	100156	89	Female	Wound (head)	ClfA_009-1	mntC-006-1	8	XKAKB	278	CC45
PFESA0362	Invasive*	NLD2	100158	63	Female	Wound (back)	ClfA_043-1	mntC-001-1	8	UJFKBPE	35	CC1
PFESA0402	Invasive	NLD2	100158	63	Female	Wound (back)	ClfA_043-1	mntC-001-1	8	UJFKBPE	35	CC1
PFESA0403	Invasive	NLD2	100158	63	Female	Wound (back)	ClfA_043-1	mntC-001-1	8	UJFKBPE	35	CC1
PFESA0288	Invasive**	NLD2	100160	72	Female	Blood	ClfA_004-1	mntC-001-7	8	VGKKAKAOQQ	5	CC30
PFESA0345	Invasive	NLD2	100160	72	Female	Shoulder punctate	ClfA_004-1	mntC-001-7	8	VGKKAKAOQQ	5	CC30
PFESA0346	Carriage	NLD2	100160	72	Female	Nasal	ClfA_004-1	mntC-001-7	8	WGKAKAOMQQ	33	CC30
PFESA0289	Invasive**	NLD2	100162	83	Female	Blood	ClfA_044-1	mntC-001-1	5	YHGFMBQBLO	1	CC8
PFESA0398	Invasive	NLD2	100162	83	Female	Wound (ankle)	ClfA_044-1	mntC-001-1	5	YHGFMBQBLO	1	CC8
PFESA0347	Carriage	NLD2	100162	83	Female	Nasal	ClfA_001-1	mntC-001-13	5	UJGFMBBBBPB	88	CC97
PFESA0297	Invasive	NLD2	100164	47	Male	Blood (2nd culture)	ClfA_009-1	mntC-006-1	8	A2N5KBEMBKB	1304	CC45
PFESA0348	Invasive	NLD2	100164	47	Male	Aneurism spurium	ClfA_009-1	mntC-006-1	8	A2NEWKBEMBKB	1304	CC45
PFESA0349	Carriage	NLD2	100164	47	Male	Nasal	ClfA_009-1	mntC-006-1	8	A2NEWKBEMBKB	1304	CC45
PFESA0350	Invasive**	NLD2	100164	47	Male	Blood (1st culture)	ClfA_002-1	mntC-001-5	5	TJMBDMGMK	45	CC5

[a]For invasive isolates, “*” signifies the primary invasive isolate recovered from a wound patient; “**” signified the primary invasive isolate from a bacteremia patient.
[b]NLD1, VUMC site; NLD2, Amphia site.
