# Supplementary material for: Evaluation of Approaches to Monitor Staphylococcus aureus Virulence Factor Expression during Human Disease
Source: PLoS One. 2015 Feb 26;10(2):e0116945. doi: 10.1371/journal.pone.0116945 (PMC4342157; doi:10.1371/journal.pone.0116945)
Supplement: S2 Table — (PDF) [file pone.0116945.s002.pdf]

Table S2. qRT-PCR Results for Nasal and Wound Samples

Subject	Swab	Sample Id	16S	clfA	mntC	cap8	tpi	icaB	isdB	cap5
100101	nasal	*M05VPF*	721	0	0	0	0	0	0	0
100101	wound	M05VJV	19091	5	4	0	0	0	0	0
100102	nasal	L01W2U	320	0	0	0	0	0	0	0
100102	nasal	N044G7	255	0	0	0	0	0	0	0
100102	wound	L01W34	337	0	0	0	0	0	0	0
100102	wound	N044GH	4	0	0	0	0	0	0	0
100103	nasal	M05VPG	1236	0	0	0	0	0	0	0
100104	nasal	L01W2V	97	0	0	0	0	0	0	0
100104	nasal	N044G8	257	0	0	0	0	0	0	0
100104	wound	L01W35	27	0	0	0	0	0	0	0
100104	wound	N044GI	29	0	0	0	0	0	0	0
100105	nasal	M05VPH	415	0	1	0	0	0	0	0
100105	wound	M05VJW	7299	3	0	0	9	0	0	0
100106	nasal	L01W2W	1497	3	0	0	0	0	0	0
100106	wound	L01W36	13850000	1455	3723	118	1081	3	49	0
100107	nasal	M05VPJ	106	0	0	0	0	0	0	0
100107	wound	M05VJX	8407	12	0	0	2	0	0	0
100108	nasal	L01W2X	782198	585	3039	0	221	0	0	0
100109	nasal	M05VPK	28	0	0	0	0	0	0	0
100110	nasal	L01W2Y	10642	1	6	0	4	0	0	0
100110	wound	L01W37	35	0	0	0	0	0	0	0
100111	nasal	M05VPL	201	0	0	0	0	0	0	0
100112	nasal	L01W2Z	12053	12	0	0	6	0	0	0
100112	nasal	N044G9	12750	3	0	0	0	0	0	0
100113	nasal	M05VPM	131	0	0	0	0	0	0	0
100114	nasal	N044GA	34	0	0	0	0	0	0	0
100114	wound	L01W38	41	0	0	0	0	0	0	0
100114	wound	N044GJ	35	0	0	0	0	0	0	0
100115	nasal	M05VPN	260	0	0	0	0	0	0	0
100117	nasal	M05VPO	2142	3	0	0	0	0	0	0
100118	nasal	L01W3E	161	0	3	0	0	0	0	0
100118	wound	N044E9	1235	0	0	0	0	0	0	0
100119	nasal	M05VPP	519	0	0	0	0	0	0	0
100120	nasal	L01W3F	14	0	0	0	0	0	0	0
100121	nasal	M05VPQ	485	0	0	8	0	0	0	0
100121	wound	M05VJY	23170	9	49	5	0	0	0	0
100122	nasal	L01W3G	114	0	0	0	0	0	0	0
100122	wound	N044EA	110871	0	1	0	0	0	0	0
100123	nasal	M05VPR	4902	0	3	0	1	0	0	0
100123	wound	M05VJZ	6840000	1533	14624	1117	1017	3	2841	0
100124	nasal	L01W3H	311	0	0	0	0	0	0	0
100124	wound	N044EB	5935	0	14	0	0	0	0	0
100125	nasal	M05VPS	313	0	0	0	0	0	0	0
100126	nasal	L01W3I	31180	71	55	0	9	0	0	0
100127	nasal	M05VPT	110361	33	156	0	32	0	0	0
100127	wound	M05VK0	40040	8	27	0	10	0	0	0
100128	nasal	L01W3J	7	0	0	0	0	0	0	0
100128	wound	N044ED	23	0	0	0	0	0	0	0
100129	nasal	M05VPU	197	0	0	0	0	0	0	0
100130	nasal	L01W3K	17	0	0	0	0	0	0	0
100130	wound	N044EE	191901	203	283	0	72	0	0	0
100131	nasal	M05VPV	1704	0	0	0	0	0	0	0
100131	wound	M05VK1	722018	386	3735	0	396	0	0	0
100132	nasal	L01W3L	3	0	0	0	0	0	0	0
100132	wound	N044EF	205	0	0	0	0	0	0	0
100133	nasal	M05VPW	368.5	0	0.5	0	0	0	0	0
100133	wound	M05VK2	6155.5	0	0.5	0	0	0	0	0

100134	nasal	L01W3M	101	0	0	0	0	0	0	0
100135	nasal	M05VPX	22	0	0	0	0	0	0	0
100136	nasal	L01W3N	55	0	0	0	0	0	0	0
100136	wound	N044EG	26513	3	18	0	3	0	0	0
100137	nasal	M05VPY	27	0	0	0	0	0	0	0
100137	wound	M05VK3	259	0	0	0	0	0	0	0
100138	nasal	N044ET	21	0	0	0	0	0	0	0
100138	wound	N044EH	347842	21	359	0	42	0	0	0
100139	nasal	M05VPZ	18	0	0	0	0	0	0	0
100140	nasal	N044EU	13	0	0	0	0	0	0	0
100142	nasal	N044EV	1055	0	0	0	0	0	0	0
100142	wound	N044FD	38176	18	4	0	0	9	0	0
100144	nasal	N044EW	454	4	0	0	0	0	0	0
100144	wound	N044FF	672	0	0	0	0	0	0	0
100146	wound	N044FG	55	0	0	0	0	0	0	0
100148	nasal	N044EY	12	0	0	0	0	0	0	0
100148	wound	N044FH	1171	0	0	0	0	0	0	0
100150	nasal	N044EZ	383	0	0	0	0	0	0	0
100150	wound	N044FI	10	0	0	0	0	0	0	0
100152	nasal	N044F0	37689	66	119	0	20	0	0	0
100154	nasal	N044F1	385	0	0	0	0	0	0	0
100154	wound	N044FJ	1771	0	2	6	1	0	0	0
100156	nasal	N044F2	48	3	0	0	0	0	0	0
100158	nasal	N044FY	460	0	0	0	0	0	0	0
100158	wound	N044FK	38607.5	0	0	0	0	0	0	0
100160	nasal	N044FZ	6540	0	17	0	0	1	0	0
100160	wound	N044FL	1866	3	0	55	5	0	0	0
100162	nasal	N044G0	4157	0	7	0	0	0	0	0
100162	wound	N044FM	189	0	0	0	0	0	0	0
100164	nasal	M05VGT	1118	0	0	0	0	0	0	0
100164	nasal	N044GV	29	1	0	0	0	0	0	0

qRT-PCR results are expressed as **relative copy numbers** measured in 1% of the RNA extracted from each sample.
